# Patient and provider preferences for long-acting TB preventive therapy

**DOI:** 10.5588/ijtldopen.24.0670

**Published:** 2025-05-12

**Authors:** M. Vermeulen, K.K. Scarsi, R. Furl, H. Sayles, M.J. Anderson, S. Valawalkar, A. Kadam, S.R. Cox, V. Mave, M. Barthwal, C. Schutz, A. Ward, J. Dountio Ofimboudem, G. Meintjes, S. Rannard, A. Owen, S. Swindells

**Affiliations:** ^1^ Department of Medicine and Institute of Infectious Disease and Molecular Medicine, University of Cape Town, Observatory, Cape Town, South Africa;; ^2^ College of Pharmacy, University of Nebraska Medical Center, Omaha, NE, USA;; ^3^ College of Medicine, University of Nebraska Medical Center, Omaha, NE, USA;; ^4^ College of Public Health, University of Nebraska Medical Center, Omaha, NE, USA;; ^5^ Johns Hopkins India, Center for Infectious Diseases in India (CIDI), Pune, India;; ^6^ Johns Hopkins Bloomberg School of Public Health, Epidemiology, Baltimore, MD, USA;; ^7^ Johns Hopkins School of Medicine, Division of Infectious Diseases, Baltimore, MD, USA;; ^8^ Dr. D. Y. Patil Medical College, Hospital and Research Centre, Department of Pulmonary Medicine, Pune, Maharashtra, India;; ^9^ Treatment Action Group, New York, NY, USA;; ^10^ Blizard Institute, Faculty of Medicine and Dentistry, Queen Mary University of London, London, UK;; ^11^ Department of Chemistry, Centre of Excellence for Long-acting Therapeutics (CELT), University of Liverpool, Liverpool, UK;; ^12^ Department of Pharmacology and Therapeutics, Centre of Excellence for Long-acting Therapeutics (CELT), University of Liverpool, Liverpool, UK.

**Keywords:** tuberculosis, prevention, long-acting, preferences, feasibility

## Abstract

**BACKGROUND:**

Tuberculosis preventive therapy (TPT) is critical for TB elimination but is underutilised. Long-acting (LA) TPT can potentially improve linkage to care, treatment adherence and outcomes.

**METHODS:**

We conducted a cross-sectional in-person survey in two high TB burden countries to evaluate preferences and concerns about LA formulations for TPT. The survey compared oral pills to LA injections, implants, and microarray patches (MAPs). A parallel online survey of healthcare providers (HCPs) in low- and middle-income countries (LMICs) assessed the perceived feasibility of implementation. Data were summarised by descriptive statistics.

**RESULTS:**

We recruited 409 patients (India, *n* = 209; South Africa, *n* = 200) and 94 HCP participants. The mean age of patients was 40 years; 65% were female, and 26% reported a history of TPT. Injectable LA-TPT was the most preferred modality, followed by pills, implants, and then MAPs. The majority (75%) expressed a strong willingness to try injectable LA-TPT. Among providers, 43% favoured injectable LA-TPT, 26% preferred oral pills, 18% implants, and 13% MAPs. Cost was a significant factor influencing HCPs’ willingness to adopt LA-TPT, while potential inefficacy and prolonged side effects were the highest concerns of patient respondents.

**CONCLUSION:**

Injectable LA-TPT may be highly acceptable and feasible if concerns surrounding cost, effectiveness, and safety are addressed.

TB preventive therapy (TPT) is the key intervention for preventing the progression of TB infection to active disease.^1–3^ However, in high TB burden settings, TPT is often underutilised.^1^ System-level barriers to implementing TPT in low- and middle-income countries (LMICs) include insufficient staffing to provide adequate adherence support,^4^ as well as limited funds, which results in drug stockouts and restricted access to shorter TPT regimens.^5^ Patient-level barriers to completing TPT are stigma related to taking daily pills, side effects, insufficient patient education on the importance of TPT, and socio-economic factors.^4,6,7^ Prolonged treatment duration results in suboptimal adherence and reduced completion rates.^8^ Shorter rifamycin-based TPT regimens have shown equivalent efficacy to 6 to 12 months of isoniazid (INH) with improved completion rates.^9,10^ However, rifamycin-based regimens are not widely available and increase the risk of drug-drug interactions, especially with antiretroviral therapy (ART).^2,11^

Long-acting (LA) TPT can potentially address some of these barriers.^12^ LA formulations have been successfully used for other prevention and treatment strategies to improve patient outcomes. The use of reversible LA contraception has been associated with higher continuation and lower rates of unintended pregnancies.^13^ In psychiatry, LA formulations have improved adherence and lower relapse- and hospitalisation rates.^14^ In HIV prevention, LA formulations have proved to be superior to daily oral pre-exposure prophylaxis for incident HIV acquisition.^15,16^ Most recently, biannual subcutaneous administration of lenacapavir was found to be effective in preventing HIV acquisition.^17^ For HIV treatment, monthly or bi-monthly injectable LA cabotegravir and rilpivirine are non-inferior to oral ART for HIV suppression.^18^

The development of LA formulations for TB is currently being explored by several groups. As part of the Unitaid-funded LONGEVITY Project, LA injectable and MAP-based delivery systems for rifapentine and INH are being developed (https://www.liverpool.ac.uk/centre-of-excellence-for-long-acting-therapeutics/longevity/). In paucibacillary mouse models, dynamic oral dosing to simulate LAI exposures of rifabutin and rifapentine had efficacious exposure profiles with equivalent bactericidal activity to one month of INH/rifapentine.^19^ Furthermore, preclinical data for the injectable were presented at the Conference on Retroviruses and Opportunistic Infections in 2024^20^ and provided proof-of-concept for LA rifapentine to achieve efficacy comparable to one month of oral rifapentine with INH in a validated mouse TPT model. An LA formulation of rifabutin using biodegradable polymers is also in development.^21^ Finally, an LA injectable bedaquiline formulation is in clinical development.^22,23^

Successful adoption of LA-TPT is dependent on acceptability by both patients and providers. We conducted a survey in two high TB burden countries to evaluate patients’ perspectives and preferences of LA-TPT and ascertain concerns relating to each administration method. We also surveyed healthcare providers to assess the feasibility of implementing LA-TPT.

## STUDY POPULATION AND METHODS

### Study design and population

We conducted the patient survey in person in India and South Africa and targeted recruitment among adults (≥18 years) representing end-users for TPT in each setting. In South Africa, we utilised convenience sampling to recruit people accessing health care at a primary health care clinic in Khayelitsha, Cape Town. In India, we recruited people who recently completed TB treatment and their household contacts during home visits conducted under the TB Aftermath study in Pune, Maharashtra.^24^ Participants were not required to have a current or historical use of TPT. Trained study staff administered the in-person survey with direct data capture using tablets. Patient surveys were available in English in South Africa, with trained study staff providing verbal translations into isiXhosa as needed. In India, the surveys were offered in English, Hindi, and Marathi.

The provider survey was administered online in English and French, targeting TB healthcare workers, programme personnel, policymakers, and Health Ministry personnel from LMICs. We recruited providers through professional TB-related mailing lists, targeted social media outreach from TB-related organisations, and coordinated with TB-related events and conferences.

Patient surveys gathered non-identifiable demographic information, medical history, and insights into participants’ experiences with various medication administration methods. Before asking questions, study staff shared illustrations and written descriptions about each formulation. Due to low familiarity with implants as a modality, we included an optional video link to demonstrate how an implant is inserted (https://www.youtube.com/watch?v=i1mL5nJps4c). The patient surveys included questions about TPT administered as pills, injectables, sub-dermal implants, and microarray patches (MAPs). Participants ranked their preferred methods of administration of TPT, perceived benefits, concerns, and willingness to try each method. We also asked parents/guardians of children about their willingness to have their children receive injectable LA-TPT.

Provider surveys included questions on country of origin and factors influencing prescribing practices of TPT, including cost, effectiveness, and safety considerations. Providers were asked about potential obstacles their country may need to address to implement LA-TPT.

Survey development was informed by prior work from this group in LA formulations for malaria and hepatitis C.^25,26^ Pilot survey data and survey administrator feedback were utilised to improve reliability, ensure proper content, and optimise consistent survey administration. (See Supplementary Data for full study instruments.).

### Statistical analysis

We collected survey data using the Research Electronic Data Capture (REDCap)) tool, which is managed by the University of Nebraska Medical Center (UNMC), Omaha, NE, USA.^27^ Data were summarised by descriptive statistics. Continuous variables with normal distribution were summarised as mean and standard deviation, while categorical variables were represented as counts and percentages. The continuous variables between the two groups were compared using analysis of variance. For categorical variables, comparisons between groups were conducted using χ^2^ or Fisher’s Exact tests. Analyses were done using Stata SE v18.0 (StataCorp LLC, College Station, TX, USA).

### Ethics

The UNMC Institutional Review Board (IRB), Omaha, NE, USA, classified the research as exempt (0304-23-EX). Sites in India (DYPV/EC/972/23) and South Africa (UCT HREC 504/2023) received local IRB approval, and patient participants provided written informed consent. A stipend was provided to all patient participants in accordance with the recommendations of the local IRB. Provider participants provided informed consent electronically and were not reimbursed for participation.

## RESULTS

### Patient survey results

A total of 409 patients completed the patient survey, 200 (49%) from South Africa and 209 (51%) from India. The mean age of patient respondents was 40 years, and 65% were female. Overall, 162 (40%) of patients reported currently taking pills for any reason, with a higher percentage among South African compared to Indian patients (69% vs. 11%; *P* < 0.001). Of those currently taking medication, 61 (38%) reported missed doses within the last 2 weeks. Among all patients, 164 (40%) reported a history of TB infection, of whom 154 (94%) experienced active TB disease. Furthermore, 103 (26%) of patients reported having taken TPT in the past, with the most common regimen being daily INH for 6–9 months (98%). In terms of comorbidities, 132 (32%) of all patients reported being diagnosed with HIV, all of whom were from South Africa (Table 1).

**Table 1.** Patient respondent demographics and clinical characteristics.

	Total	South Africa	India	*P*-value
	(*n* = 409)	(*n* = 200)	(*n* = 209)	
	*n* (%)	*n* (%)	*n* (%)	
Age in years, mean ± SD	40.4 ± 13.1	39.2 ± 10.8	41.6 ± 14.9	<0.001^*^
Race				
Black African	198 (49)	198 (99)	0 (0)	
Asian	207 (51)	0 (0)	207 (100)	
Other	3 (1)	2 (1)	1 (0)	<0.001^*^
Sex				
Male	142 (35)	47 (24)	95 (45)	
Female	267 (65)	153 (76)	114 (55)	<0.001^†^
Previous TB				
Yes	164 (40)	67 (34)	97 (46)	
No	243 (59)	132 (66)	111 (53)	
Don’t know	2 (0)	1 (0)	1 (0)	0.013^*^
Specify previous TB				
Active TB disease	154 (94)	64 (96)	90 (93)	0.529^*^
Latent TB infection	10 (6)	3 (4)	7 (7)	
Currently taking any medication in the pill form				
Yes	162 (40)	138 (69)	24 (11)	
No	247 (60)	62 (31)	185 (89)	<0.001^†^
Number of pills per day				
0 or <1	10 (6)	0 (0)	10 (42)	
1–2	96 (59)	85 (62)	11 (46)	
3–5	46 (28)	43 (31)	3 (12)	
6–9	8 (5)	8 (6)	0 (0)	
>9	2 (1)	2 (1)	0 (0)	<0.001^*^
Last reported missed dose				
Within the past week	27 (17)	26 (19)	1 (4)	
1–2 weeks ago	34 (21)	28 (20)	6 (25)	
3–4 weeks ago	10 (6)	10 (7)	0 (0)	
1–3 months ago	18 (11)	17 (12)	1 (4)	
>3 months ago	12 (7)	11 (8)	1 (4)	
Never skipped	61 (38)	46 (33)	15 (62)	0.070^*^
Previous medication by injection				
Yes	256 (63)	155 (78)	101 (48)	
No	149 (36)	44 (22)	105 (50)	
Prefer not to answer/don’t know	4 (1)	1 (1)	3 (1)	<0.001^*^
Previous TPT use				
Yes	103 (26)	54 (27)	49 (25)	
No	289 (73)	145 (72)	144 (74)	
Unsure/don’t know	2 (1)	1 (0)	1 (1)	0.865^*^

* Fisher’s exact test.

† χ^2^ test.

SD = standard deviation; TPT = TB preventive therapy.

**Figure 1.**
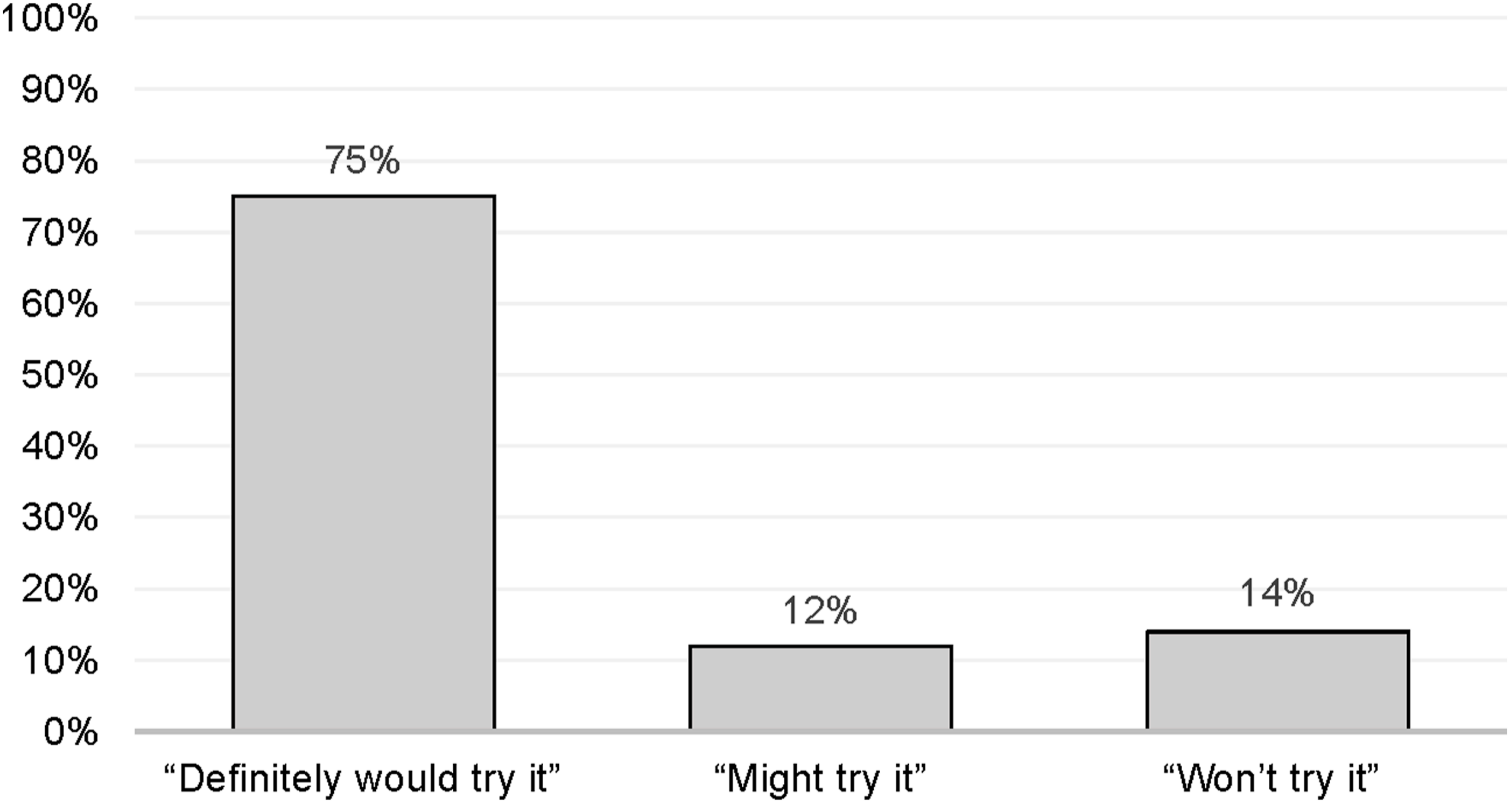
Patient respondent willingness to try injectable TPT. TPT = TB preventive therapy.

The majority of patients expressed a willingness to try injectable TPT, with 304 (75%) indicating they ‘definitely would try it,’ 48 (12%) ‘might try it,’ and only 55 (14%) ‘will not try it’ (Figure 1). No significant difference in willingness based on sex or age demographics was observed. Patients with no prior history of TPT showed greater enthusiasm for injectable TPT than those with a history of oral TPT use (71% vs. 29%; *P* = 0.129). Among all respondents, 256 (63%) had prior experience with medication being administered by injection (Table 1). Patients with prior use of injectables for any other medical reason demonstrated a significantly higher willingness to try injectable TPT than those without prior injectable use (73% vs. 27%; *P* < 0.001). Most patients (*n* = 277, 68%) regarded injections as the strongest/most effective method, followed by pills (*n* = 76, 19%), implants (*n* = 36, 9%), and MAPs (*n* = 19, 5%).

**Figure 2.**
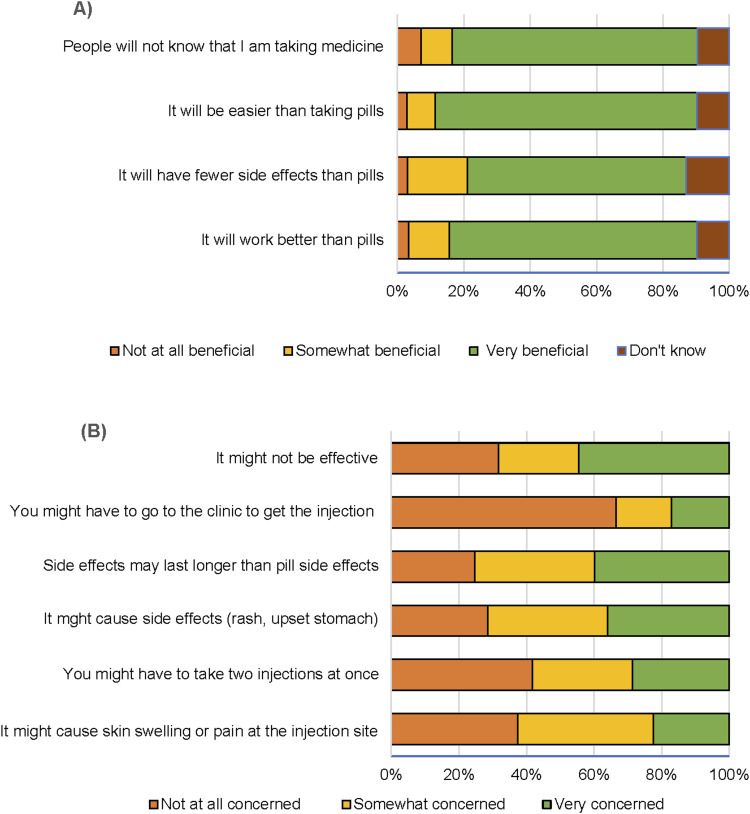
**A)** Perceived benefits of injectable TPT among patient respondents; **B)** concerns about injectable TPT among patient respondents. TPT = TB preventive therapy.

Respondents indicated that injectable TPT would be ‘very beneficial’ due to ease of administration (*n* = 304, 79%) and perceived efficacy (*n* = 290, 75%) compared to pills (Figure 2A). However, (*n* = 169, 45%) of patients reported being ‘very concerned’ about the injection being ineffective and (*n* = 155, 40%) were concerned that side effects may last longer than side effects from pills (Figure 2B). Most respondents (*n* = 298, 77%) were ‘very likely’ to try an injectable that is given once a month for 2 months, and (*n* = 291, 75%) preferred once a month for 3 months. However, (*n* = 186, 93%) South Africans preferred one single injection.

Only 156 (41%) of patients expressed willingness to try the implant, with a disparity observed between South African and Indian respondents (64% vs. 15%). The primary concerns reported by patients were ongoing pain after implant insertion (*n* = 222, 70%) and concern about the efficacy of the implant (*n* = 217, 68%). Related to MAPs, 198 (48%) of patients were willing to receive a MAP; South African respondents were more amenable to receiving MAPs compared to their Indian counterparts (72% vs. 25%, respectively). The main concerns with MAPs were concerns about lack of efficacy (*n* = 197, 60%) and the possibility that side effects may last longer than pills (*n* = 187, 58%).

Among respondents who identified themselves as the parent or guardian of children under the age of 12 years (*n* = 176, 43%), 132 (75%) expressed willingness to have their child receive an injection for TPT. This was significantly higher among South African respondents than Indian respondents (93% versus 51%, respectively). Concerns regarding injectable TPT were common, with 90 (53%) of parents/guardians being ‘very worried’ about potential side effects such as rash or upset stomach. Additionally, 101 (59%) of parents/guardians were concerned that side effects from an injection may last longer than from a pill. For parents/guardians of children older than 12 years, 127 (79%) reported a willingness to have their children receive injectable TPT, with higher acceptance among South Africans compared to Indians (95% vs. 54%, respectively). The most common concern among this group was side effects lasting longer (*n* = 83, 53%).

### Provider survey results

The provider survey was completed by 94 providers from 23 LMICs and two high-income countries. Among providers, 14 (15%) identified as TB treatment prescribers, while 67 (74%) were involved with developing TB treatment/prevention policy. Most providers, 69 (77%) had ≥5 years of experience with TPT, with 40% reporting having prescribed TPT for ≥100 patients in the past 12 months (Table 2). Most providers (92%) confirmed the availability of a national guideline for TPT in their respective countries, with daily INH for 6–9 months being the most commonly prescribed regimen. Of the 38 (46%) providers who prescribed TPT, 16 (43%) practised in urban areas and 15 (41%) in rural areas.

**Table 2.** Provider respondent characteristics.

	Provider
	(*n* = 94)
	*n* (%)
WHO Region	
African	50 (53)
Western Pacific	11 (12)
South-East Asian	5 (5)
Regions of America	3 (3)
European	2 (2)
Not specified	22 (23)
Roles in TB care	
General provider, occasionally provide care for TB	25 (27)
Provide specialist care for TB	14 (15)
Train other providers on TB care and treatment	32 (34)
Develop and/or implement guidelines for TB prevention and treatment	35 (37)
Conduct research on TB prevention and treatment	19 (20)
Other	18 (19)
Experience with TPT, years	
<5	21 (23)
5–10	33 (37)
10–20	21 (23)
>20	15 (17)
Prescribe TPT	
Yes	38 (46)
No	39 (48)
Unsure	5 (6)
Practice settings for prescribing TPT	
Hospitals	18 (19)
Community-based centres	18 (19)
Outpatient clinics	14 (15)
Prisons	6 (6)
Specialised centres	3 (3)
Mobile clinics	3 (3)

TPT = TB preventive therapy.

**Figure 3.**
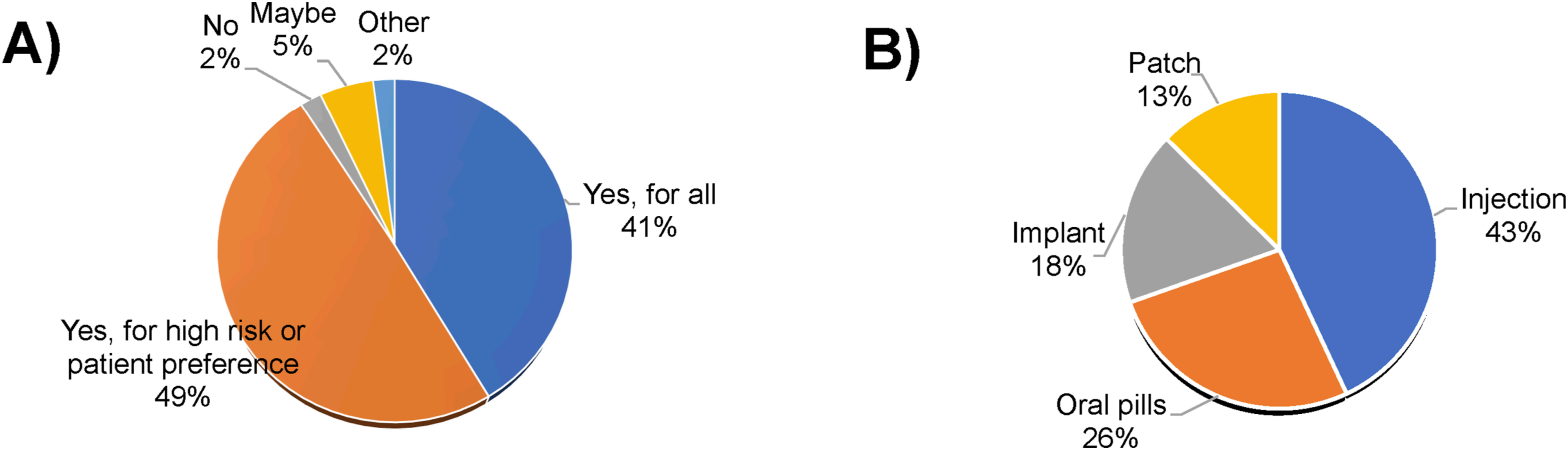
**A)** Provider acceptability – ‘If approved, and efficacy, safety, and cost were the same would you prescribe a long-acting medication for TB prevention rather than oral medication?’ **B)** Provider preference – ‘If efficacy, safety, and cost were the same, which modality would you most prefer to prescribe?’

Among 94 providers, 90% expressed willingness to prescribe LA-TPT if efficacy, safety, and cost were the same as oral pills, with 41% reporting they would prescribe for all patients. The other 49% would limit prescription to patients with HIV co-infection, other comorbidities, or patients at high risk for suboptimal adherence due to socio-economic factors or being from a marginalised group (Figure 3A). The preferred formulation among providers was injectable TPT (*n* = 34, 43%) (Figure 3B). Most providers (*n* = 57, 75%) indicated they would prescribe an LA formulation if it were equal to, or less expensive than, oral medications. The factors influencing a prescriber’s decision to prescribe LA-TPT, defined as ‘moderate to very important’, included improved adherence (*n* = 81, 99%) and better efficacy (*n* = 79, 98%). Overall, implementing injectable TPT was considered feasible by most providers (*n* = 65, 81%).

In the opinion of the providers, improved patient satisfaction or quality of life (*n* = 76, 97%) and better efficacy (*n* = 76, 94%) were ‘moderately to very likely’ to influence governments or health systems to introduce LA-TPT. Providers identified potential obstacles to the introduction of LA-TPT, with a majority citing the cost of drugs (*n* = 72, 86%) and concern about side effects (*n* = 63, 79%).

## DISCUSSION

We found high levels of acceptability and perceived feasibility of LA-TPT among patients and providers. We observed a strong preference for injectable TPT, particularly among patients with prior experience with injections. The strong willingness to adopt injectable TPT suggests that familiarity may be key to its acceptability. Patients and providers highlighted ease of administration and perceived efficacy as key advantages of injectable TPT. Among providers, injectable LA-TPT was the preferred modality, provided cost, safety, and efficacy were the same as oral pills. Providers identified cost as a potential barrier to implementation by national governments. Patients’ preferences for dosing intervals differed between countries, with South Africans preferring a single administration. Indian participants preferred once-a-month administration for 2–3 months. While not explored, a single administration may be more convenient, requiring less time at healthcare facilities, while multiple doses offer more opportunities for healthcare access and doctor consultations for side effects. Among parents or guardians, injectable TPT was an acceptable method of administration for their children, regardless of age. However, we observed a higher willingness among South African compared to Indian parent/guardian respondents. Oral TPT regimens have been criticised for being unpalatable and not child-friendly;^28^ therefore, LA-TPT presents a promising alternative. Caregivers have identified high pill burden, dosing frequency, and prolonged treatment durations as key factors that influence TPT preference.^29,30^

Preference data on LA TPT are lacking; however, data on HIV prevention preferences show high acceptability of LA Pre-exposure prophylaxis (LA PrEP), especially in high-risk regions.^31,32^ Among key populations, LA PrEP, is well accepted, though administration preferences vary.^33,34^

Our study found differences in the ranking of preferred administration methods for LA-TPT across countries. South Africans ranked implants as their second preference, likely due to greater familiarity with implants as contraceptives, while Indians ranked pills second. Surveys about LA ART in youth ages 13–24 found higher acceptability for implantable ART for HIV prevention in female respondents who had experience with implantable contraception.^35^ Conversely, South African respondents’ least preferred method was oral pills. Notably, a higher percentage of South African respondents reported currently using pills, which were likely ART, given that one-third of respondents reported living with HIV. Chronic pill use can result in pill fatigue and aversion, which are common barriers to adherence.^36^ Participants reported forgetting to take pills for various reasons as a common reason for non-adherence, which is reported by other studies.^28^ One-fifth of respondents also admitted to feeling embarrassed about taking oral medication, speaking to the possible stigma attached to taking chronic pills.

Our study has limitations. The findings may not be representative of other countries, as the patient survey was only conducted in two countries. The patient survey was available in three written languages in India but only in English in SA. Study staff translated questions into isiXhosa as necessary, and this process may have introduced variability in interpretation. Some survey questions may have been challenging for patients to understand. To mitigate these issues, all personnel were trained to ensure a standardised survey process and to provide clarification as needed. Providers who completed the survey did so voluntarily and may have had a particular interest in LA-TPT, leading to an overestimation of acceptability. We had a relatively small sample size of providers, which may limit the generalizability of our findings; however, most of the respondents were from LMICs, aligning with the target demographic of an LA-TPT product.

To our knowledge, this is the first survey on preferences for LA-TPT. Future research should involve more comprehensive qualitative studies to better understand patient and provider preferences. Patient-centred care, the first pillar of the WHO’s End TB strategy, has prompted an exploration of patient preferences.^37^ Injectable TPT may be highly acceptable and feasible if cost and safety concerns are addressed. Therefore, the development and implementation of effective and safe LA-TPT should be expedited to expand treatment options and potentially improve health outcomes in high-burden TB settings.

## Supplementary Material



## References

[1] World Health Organization. Global tuberculosis report, 2024. Geneva, Switzerland: WHO, 2024.

[2] World Health Organization. WHO consolidated guidelines on tuberculosis. Module 1: prevention – tuberculosis preventive treatment, second edition. Geneva, Switzerland: WHO, 2024. 39298638

[3] Migliori GB, Clinical standards for the diagnosis, treatment and prevention of TB infection. Int J Tuberc Lung Dis*.* 2022;26(3):190–205. 35197159 10.5588/ijtld.21.0753 PMC8886963

[4] Gust DA, Risk factors for non-adherence and loss to follow-up in a three-year clinical trial in Botswana. PLoS One*.* 2011;6(4):e18435. 21541021 10.1371/journal.pone.0018435 PMC3081815

[5] Surie D, Policies, practices and barriers to implementing tuberculosis preventive treatment—35 countries, 2017. Int J Tuberc Lung Dis*.* 2019;23(12):1308–1313. 31931915 10.5588/ijtld.19.0018

[6] Boffa J, When prevention is dangerous: perceptions of isoniazid preventive therapy in KwaZulu-Natal, South Africa. Public Health Action*.* 2019;9(1):24–31. 30963039 10.5588/pha.18.0040 PMC6436494

[7] Baloyi DP, Reasons for poor uptake of TB preventive therapy in South Africa. Public Health Action*.* 2022;12(4):159–164. 36561901 10.5588/pha.22.0030 PMC9716815

[8] Macaraig MM, Improved treatment completion with shorter treatment regimens for latent tuberculous infection. Int J Tuberc Lung Dis*.* 2018;22(11):1344–1349. 30355415 10.5588/ijtld.18.0035 PMC6309173

[9] Swindells S, One month of rifapentine plus isoniazid to prevent HIV-related tuberculosis. N Engl J Med*.* 2019;380(11):1001–1011. 30865794 10.1056/NEJMoa1806808 PMC6563914

[10] Sterling TR, Three months of rifapentine and isoniazid for latent tuberculosis infection. N Engl J Med*.* 2011;365(23):2155–2166. 22150035 10.1056/NEJMoa1104875

[11] Maartens G, Effectiveness and safety of antiretrovirals with rifampicin: crucial issues for high-burden countries. Antivir Ther*.* 2009;14(8):1039–1043. 20032533 10.3851/IMP1455

[12] Ammerman NC, Potential impact of long-acting products on the control of tuberculosis: preclinical advancements and translational tools in preventive treatment. Clin Infect Dis*.* 2022;75(Suppl 4):S510–S516. 36410384 10.1093/cid/ciac672 PMC10200320

[13] Winner B, Effectiveness of long-acting reversible contraception. N Engl J Med. 2012;366(23):1998–2007. 22621627 10.1056/NEJMoa1110855

[14] Kaplan G, Impact of long-acting injectable antipsychotics on medication adherence and clinical, functional, and economic outcomes of schizophrenia. Patient Prefer Adherence*.* 2013;7:1171–1180. 24265549 10.2147/PPA.S53795 PMC3833623

[15] Delany-Moretlwe S, Cabotegravir for the prevention of HIV-1 in women: results from HPTN 084, a phase 3, randomised clinical trial. Lancet*.* 2022;399(10337):1779–1789. 35378077 10.1016/S0140-6736(22)00538-4 PMC9077443

[16] Landovitz RJ, Cabotegravir for HIV prevention in cisgender men and transgender women. N Engl J Med*.* 2021;385(7):595–608. 34379922 10.1056/NEJMoa2101016 PMC8448593

[17] Bekker LG, Twice-yearly lenacapavir or daily F/TAF for HIV prevention in cisgender women. N Engl J Med*.* 2024;391(13):1179–1192. 39046157 10.1056/NEJMoa2407001

[18] Swindells S, Long-acting cabotegravir and rilpivirine for maintenance of HIV-1 suppression. N Engl J Med. 2020;382(12):1112–1123. 32130809 10.1056/NEJMoa1904398

[19] Chang YS, Using dynamic oral dosing of rifapentine and rifabutin to simulate exposure profiles of long-acting formulations in a mouse model of tuberculosis preventive therapy. Clin Infect Dis*.* 2023;67(7):985–994. 10.1128/aac.00481-23 PMC10353356 37338374

[20] Henry Pertinez NC, Long-acting injectable rifapentine with activity in a mouse model of tuberculosis preventive therapy. 30^th^ *CROI*; Denver, CO, USA; February 19–22, 2023.

[21] Kim M, A long-acting formulation of rifabutin is effective for prevention and treatment of *Mycobacterium tuberculosis*. *Nat Commun*. 2022;13(1):2345–2352. 35941109 10.1038/s41467-022-32043-3 PMC9360445

[22] Kaushik A, Efficacy of long-acting bedaquiline regimens in a mouse model of tuberculosis preventive therapy. Am J Respir Crit Care Med*.* 2022;205(5):570–579. 34939891 10.1164/rccm.202012-4541OC

[23] Chihota V, Tuberculosis preventive treatment in high TB-burden settings: a state-of-the-art review. Drugs*.* 2024;84(2):153–169. 10.1007/s40265-024-02131-3 PMC11802714 39733063

[24] Cox SR, Tuberculosis (TB) aftermath: study protocol for a hybrid type I effectiveness-implementation non-inferiority randomized trial in India comparing two active case-finding strategies. Trials*.* 2022;23(1):145–158. 35932062 10.1186/s13063-022-06503-6 PMC9354295

[25] Furl R, Preferences and feasibility of long‐acting technologies for the treatment of hepatitis C virus: A survey of patients in diverse low‐ and middle‐income countries. J Viral Hepat. 2025;32(4):e14031. 39545599 10.1111/jvh.14031 PMC11883452

[26] Gupta N, Preferences and feasibility of long-acting technologies for treatment of hepatitis C virus in low- and middle-income countries. J Viral Hepat*.* 2024;31(5):221–232. 38545826 10.1111/jvh.13921

[27] Harris PA, The REDCap consortium: building an international community of software platform partners. J Biomed Inform*.* 2019;95:103208. 31078660 10.1016/j.jbi.2019.103208 PMC7254481

[28] Yuen CM, Toward patient-centered tuberculosis preventive treatment: preferences for regimens and formulations in Lima, Peru. BMC Public Health*.* 2021;21(1):121–133. 33430823 10.1186/s12889-020-10098-5 PMC7802335

[29] Hirsch-Moverman Y, Tuberculosis preventive treatment preferences among caregivers of children in Lesotho: a pilot study. Int J Tuberc Lung Dis*.* 2018;22(8):858–862. 29991393 10.5588/ijtld.17.0809 PMC8422791

[30] Strauss M, TB preventive therapy preferences among children and adolescents. Int J Tuberc Lung Dis*.* 2023;27(7):520–529. 37353873 10.5588/ijtld.22.0645 PMC10321362

[31] Tolley EE, Acceptability of a long-acting injectable HIV prevention product among US and African women: findings from a phase 2 clinical trial (HPTN 076). J Int AIDS Soc*.* 2019;22(10):e25408. 31651098 10.1002/jia2.25408 PMC6813716

[32] Ngure K, PrEP formulation preferences of PrEP-experienced African women for HIV prevention. J Acquir Immune Defic Syndr*.* 2021;88(4):e30–e32. 34446676 10.1097/QAI.0000000000002793 PMC8556312

[33] Levy ME, Willingness of men who have sex with men in Washington, DC to use long-acting injectable HIV pre-exposure prophylaxis. PLoS One*.* 2017;12(8):e0183521. 28827821 10.1371/journal.pone.0183521 PMC5565177

[34] Montgomery ET, Long-acting injection and implant preferences and trade-offs for HIV prevention among South African male youth. J Acquir Immune Defic Syndr*.* 2021;87(3):928–936. 33633031 10.1097/QAI.0000000000002670 PMC8192422

[35] Weld ED, Interest of youth living with HIV in long-acting antiretrovirals. J Acquir Immune Defic Syndr*.* 2019;80(2):190–197. 30418298 10.1097/QAI.0000000000001896 PMC6331217

[36] Scarsi KK, The promise of improved adherence with long-acting antiretroviral therapy: what are the data? J Int Assoc Provid AIDS Care. 2021;20:23259582211009011. 33902356 10.1177/23259582211009011 PMC8082990

[37] Myburgh H, A scoping review of patient-centred tuberculosis care interventions: gaps and opportunities. PLoS Glob Public Health*.* 2023;3(2):e0001357. 36963071 10.1371/journal.pgph.0001357 PMC10021744

