# Supplementary material for: Patient and provider preferences for long-acting TB preventive therapy
Source: IJTLD Open. 2025 May 12;2(5):276–83. doi: 10.5588/ijtldopen.24.0670 (PMC12068448; doi:10.5588/ijtldopen.24.0670)
Supplement: Supplementary file 1 [file ijtldopen24-0670_supplementarydata1.pdf]

http://dx.doi.org/10.5588/ijtldopen.24.0670

Patient and provider preferences for long-acting TB preventive therapy

LONGEVITY TB Patient Survey

READ TO THE PARTICIPANT:

Tuberculosis (TB) is a bacterial infection that is spread thought droplets in the air when a person with TB disease coughs, sneezes, or spits. People with TB infection sometimes have no symptoms and sometimes do not know they have it (this is called latent TB infection). Almost ¼ of people in the world have TB infection, but only a portion develop TB disease (when people become ill with TB, also called active TB disease). Some people have a higher risk of developing active TB disease, for example if they are older, have HIV, or have other conditions that weaken the immune system. In this survey, we are discussing treatments to prevent latent TB infection from progressing to active TB disease. This is called TB preventive treatment.

Current TB preventive treatments involve taking daily pills for one to nine months. We are working on easier new ways to give TB preventive treatment. We want your input to understand the best way. Your answers won't change anything about your current care.

Tablet User Name (do NOT edit)

____

1.) In which country do you reside?

◯ South Africa
◯ India

2.) How would you describe the area you reside?

◯ Urban
◯ Rural
◯ Other

Please specify the area where you reside:

____

3.) This is a list of terms that people use to describe their sex, please check a term that applies to you.

◯ Male
◯ Female
◯ I prefer not to answer
◯ Transgender male-to-female
◯ Transgender female-to-male
◯ Other

Please specify your sex description:

____

4a.) Are you currently breast feeding?

◯ Yes
◯ No

4b.) To your knowledge, are you now pregnant?

◯ Yes
◯ No
◯ Don't know / Not sure

5.) What is your race/ethnicity, please check all terms that apply to you.

☐ Black African
☐ Asian
☐ Hispanic or Latinx (regardless of race)
☐ Multiple races
☐ White (non-Hispanic)
☐ None of these terms apply
☐ I prefer not to answer

Please describe your race/ethnicity: __________________________________

6.) In what year were you born? __________________________________

7.) Are you taking any medication in the pill form now for any reason?

◯ Yes
◯ No

7a.) Approximately how many pills do you take each day?

◯ 0 or < 1
◯ 1-2
◯ 3-5
◯ 6-9
◯ >9

7b.) When was the last time you missed any of your medicines?

◯ Within the past week
◯ 1-2 weeks ago
◯ 3-4 weeks ago
◯ 1-3 months ago
◯ More than 3 months ago
◯ I never skip medications

7c.) Please tell us if any of the following are reasons why you might miss doses of daily pills (choose all that apply to you):

☐ Forget to take them
☐ Lose them
☐ Don't have a place to live
☐ Work schedule
☐ Cost
☐ Other reason(s)

Please specify: __________________________________

7d.) Do you ever feel ashamed or embarrassed to be seen taking pills, or worry that people might find out that you are taking them?

◯ Yes
◯ No
◯ Prefer not to answer
◯ Don't know

8.) Have you ever been told you have latent TB infection or active TB disease?

◯ Yes
◯ No
◯ I don't know

8a.) Please specify:

◯ Latent infection
◯ Active disease

9.) Have you ever taken TB preventive treatment (please do NOT include treatment for illness from active TB disease)?

◯ Yes
◯ No
◯ I don't know

9a.) Which TB preventive treatment did you take?

◯ 6 - 9 months of daily isoniazid
◯ 4 months of daily rifampin
◯ 3 months of daily isoniazid with rifampin
◯ 3 months of weekly rifapentine with isoniazid
◯ 1 month of daily rifapentine with isoniazid
◯ Other
◯ I don't know

Please specify: ______________________________

9b.) Did you complete treatment?

◯ Yes
◯ No
◯ I don't know

10.) Please note if you have any of the following conditions (you can check more than one):

☐ HIV disease
☐ Diabetes
☐ Tobacco use

READ TO THE PARTICIPANT:

We now want to ask you about 3 new ways to take medication for TB preventive treatment. We want your thoughts on this even if you do not have latent TB infection, have already been treated for latent TB infection in the past, or are currently receiving TB preventive treatment.

The first new way to take TB preventive treatment would be an injection into a muscle. We are not asking about vaccinations. Some examples of injection medications are: penicillin or antibiotic shots for infections, injections for pain, insulin or birth control like Depo-Provera

AN INJECTION LIKE THIS INTO YOUR BUTTOCKS, THIGH, OR ARM

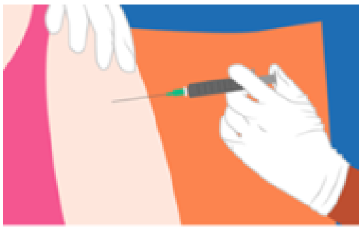

11.) Have you ever taken medications by injection instead of taking them by mouth?

◯ Yes
◯ No
◯ Prefer not to answer
◯ I don't know

12.) If you were offered TB prevention treatment by injection instead of oral pills, would you try it?

◯ Definitely would try it
◯ Might try it
◯ Definitely would not try it

13.) When you think about getting an injection for TB preventive treatment, how concerned are you that:

	Not at all concerned	Somewhat concerned	Very concerned
It might cause skin swelling or pain at the place where the injection was given	◯	◯	◯
You might have to have 2 injections at once	◯	◯	◯
It might cause side effects (rash, upset stomach)	◯	◯	◯
The side effects might last longer than pill side effects	◯	◯	◯
You might have to go to the clinic to get the injection	◯	◯	◯
It might not be effective	◯	◯	◯

14.) When you think about getting an injection for TB preventive treatment, how beneficial do you consider each of the following to be?

	Not at all beneficial	Somewhat beneficial	Very beneficial	Don't know
It will work better than pills	◯	◯	◯	◯
It will have less side effects than pills	◯	◯	◯	◯
It will be easier than taking pills	◯	◯	◯	◯
People will not know that I am taking medicine	◯	◯	◯	◯

15.) How likely would you be to try this new injectable method of TB preventive treatment if it meant you were required to come to the clinic or doctor's office:

	Not at all likely	Somewhat likely	Very likely	Don't know
...once only	◯	◯	◯	◯
...once every two weeks	◯	◯	◯	◯
...once a month for two months	◯	◯	◯	◯
...once a month for three months	◯	◯	◯	◯

READ TO THE PARTICIPANT:

The second new way to take TB preventive Treatment would be an IMPLANT. ....

AN IMPLANT LIKE THIS PUT SURGICALLY UNDER YOUR SKIN

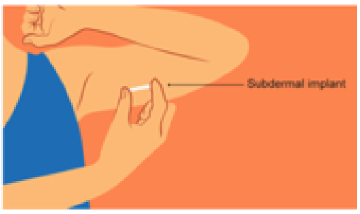

TO THE SURVEY ADMINISTRATOR:

Ask the respondent if they would like more information about the implant option. If yes, show the brief video about contraceptive implant insertion downloaded on your iPad (with permission.)

16.) In the past, have you ever had an implant?

◯ Yes
◯ No
◯ Refused
◯ Don't know

17.) If an implant worked just as well as taking pills, would you be willing to receive an implant for TB preventive treatment if needed?

◯ Yes
◯ No
◯ Refused
◯ Don't know

18.) When you think about getting an implant how concerned are you that:

	Not at all concerned	Somewhat concerned	Very concerned
It might cause a scar	◯	◯	◯
It might have to be taken out	◯	◯	◯
It might be visible to others	◯	◯	◯
It might cause side effects (rash, upset stomach)	◯	◯	◯
Side effects might last longer than for a pill	◯	◯	◯
It might not be effective	◯	◯	◯
It might cause pain with insertion	◯	◯	◯
It might cause ongoing pain after insertion	◯	◯	◯

READ TO THE PARTICIPANT:

A third new way to take TB preventive treatment would be a microneedle patch. This adhesive patch is like a sticker you put on your skin, usually your upper arm, which contains tiny needles that slowly release medicine (see photo).

A MICRONEEDLE PATCH STUCK ONTO YOUR SKIN

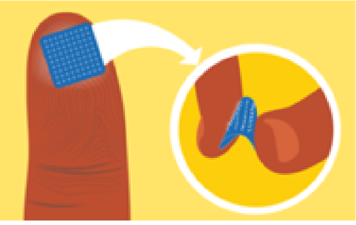

19.) If a microneedle patch worked just as well as taking pills, would you be willing to receive a microneedle patch for TB preventive treatment if needed?

◯ Yes
◯ No
◯ Refused
◯ Don't know

20.) When you think about taking the medication by the Microneedle patch how concerned are you that it:

	Not at all concerned	Somewhat concerned	Very concerned
Might cause a rash	◯	◯	◯
Might cause pain	◯	◯	◯
Might not be effective	◯	◯	◯
Might cause other side effects (headache, upset stomach)	◯	◯	◯
Side effects might last longer than for a pill	◯	◯	◯
It might be visible to others	◯	◯	◯

READ TO THE PARTICIPANT:

We have discussed four different TB preventive treatment options...

Current available options:
Daily pills

New long-acting treatment options:
Injection(s)
Implant
Microneedle patch

A.) PILLS

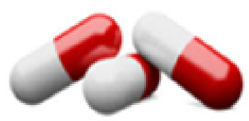

B.) INJECTION(s)

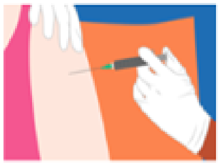

C.) IMPLANT

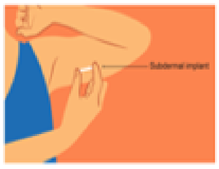

D.) MICRONEEDLE PATCH

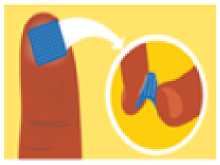

21.) Which method do you feel is the strongest/most effective method of treatment?

◯ Pills
◯ Injections
◯ Implant
◯ Microneedle patch

22.) Imagine all of these methods work just as well, cost the same, and have the same chance of side effects. Please rank your preference in ways of taking medicine in order:

	Rank #1	Rank #2	Rank #3	Rank #4
Pills	◯	◯	◯	◯
Injections	◯	◯	◯	◯
Implant	◯	◯	◯	◯
Microneedle patch	◯	◯	◯	◯

23.) Are you a parent or guardian of a child who is under 12 years old?

◯ Yes
◯ No

23a.) If the new injection for TB preventive treatment was available for children younger than 12 years old, would you have your child receive it?

◯ I definitely would have my child receive
◯ I might have my child receive it
◯ I definitely would not have my child receive it

23b.) When you think about getting an injection for TB preventive treatment for your child who is under 12 years old, how worried are you that:

	Not at all worried	Somewhat worried	Very worried
They will have to get an injection	◯	◯	◯
It will be painful	◯	◯	◯
It might cause side effects (rash, upset stomach)	◯	◯	◯
The side effects might last longer than pill side effects	◯	◯	◯
I will have to bring them to the clinic to get the injection	◯	◯	◯

24.) Are you a parent or guardian of a child who is 12 years or older?

◯ Yes
◯ No

24a.) If the new injection for TB preventive treatment was available for children aged 12 years or older, would you have your child receive it?

◯ I definitely would have my child receive
◯ I might have my child receive it
◯ I definitely would not have my child receive it

24b.) When you think about getting an injection for TB preventive treatment for your child who is 12 years or older, how worried are you that:

	Not at all worried	Somewhat worried	Very worried
They will have to get an injection	◯	◯	◯
It will be painful	◯	◯	◯
It might cause side effects (rash, upset stomach)	◯	◯	◯

Powered by REDCap

The side effects might last longer than pill side effects	◯	◯	◯
I will have to bring them to the clinic to get the injection	◯	◯	◯

25.) Have you ever had to pay for TB preventive treatment?

◯ Yes
◯ No

25a.) Think for a moment about how much you currently pay for your TB preventive treatment, or how much you have paid in the past. How likely would you be to try this new way of taking TB preventive treatment if the cost of this new way of taking medicine is: (Please mark one box in each row).

	Definitely would try it	Might try it	Definitely would not try it
... much less than you pay now	◯	◯	◯
... a little less than you pay now	◯	◯	◯
... the same amount you pay now	◯	◯	◯
... a little more than you pay now	◯	◯	◯
... much more than you pay now	◯	◯	◯

26.) Any other comments (or reasoning if survey is incomplete)?

Powered by REDCap

Provider Attitudes to, and Interest in, Long-Acting Formulations of Tuberculosis Treatment - UNMC IRB #0304-23-EX

Dear Survey Participant:

Thank you for participating in this provider survey for our research study entitled "Patient and Provider Attitudes to, and Interest in, Long-Acting Formulations of Tuberculosis Treatment". This is a voluntary online survey for program and health care providers about the prevention of tuberculosis.

Survey Background:

"Long-acting" medications refer to formulations of medications that prolong the effect of the medication. These medications can be administered through different routes such as injections, implants, patches, or other technologies. Long-acting medications are commonly used to improve adherence and treatment outcomes for a range of indications, including contraception, HIV treatment and prevention, and psychiatric conditions.

There are currently several long-acting formulations under development for the prevention of tuberculosis (TB). This work is being conducted as part of the LONGEVITY project, funded by UNITAID, specifically targeting implementation in Low- and Middle-Income Countries (LMICs). As part of the development of these medications, we are conducting this survey to understand the preferences of program and clinical providers.

Survey Objectives:
This survey assesses provider and policymaker perspectives on long-acting TB preventive treatment.

In this survey, we will ask about the following potential formulations for a long-acting medication for TB preventive treatment:

Injection: A medicine is injected into the muscle tissue. Intramuscular administrations are generally given in the arm, thigh, hip, or buttocks (see injection illustration in survey).

Implant: A small rod (approximately 4 cm x 2 mm) containing the medication that is typically inserted under the skin of the upper arm (minor procedure with incision). The implant then either slowly dissolves and requires no removal or is removed by a clinician (minor procedure with incision) (see implant illustration in survey).

Patch: Microneedle patches are devices that allow a medication to be delivered through the skin. A patch is typically a 4x4cm skin-colored adhesive placed anywhere on the skin and is applied and removed by the patient (see patch illustration in survey).

Confidentiality & Intended Use:

The total estimated time for the survey is 15 minutes. You will not be asked for any identifying information or for any information regarding individual patients. You may choose to stop the survey at any time. You may change your responses prior to submitting the survey.

Participant Benefits:

There is no direct benefit to you as a result of participation in this study.

Findings from this study will be used to inform development of long-acting medications for the prevention of TB in LMICs.

Study contact:
Dr. Susan Swindells
University of Nebraska Medical Center
sswindells@unmc.edu

Survey Instructions:

Please read all questions carefully and respond to the best of your knowledge regarding patients in your practice (if you are a prescribing clinician) or TB prevention in your country (if you are not a prescribing clinician).

Module Selection (select one):

◯ I am a TB treatment prescriber
◯ I am not a TB treatment prescriber but I am involved in TB prevention and treatment policies in my country
◯ Other

Please describe: ______________________________

Country:

◯ Afghanistan
◯ Albania
◯ Algeria
◯ Andorra
◯ Angola
◯ Antigua and Barbuda
◯ Argentina
◯ Armenia
◯ Austria
◯ Australia
◯ Azerbaijan
◯ Bahrain
◯ Bangladesh
◯ Barbados
◯ Belarus
◯ Belgium
◯ Belize
◯ Benin
◯ Bhutan
◯ Bolivia
◯ Bosnia and Herzegovina
◯ Botswana
◯ Brazil
◯ Brunei
◯ Bulgaria
◯ Burkina Faso
◯ Burundi
◯ Cabo Verde
◯ Cambodia
◯ Cameroon
◯ Canada
◯ Central African Republic
◯ Chad
◯ Channel Islands
◯ Chile
◯ China
◯ Colombia
◯ Comoros
◯ Congo
◯ Costa Rica
◯ Côte d'Ivoire
◯ Croatia
◯ Cuba
◯ Cyprus
◯ Czech Republic
◯ Denmark
◯ Djibouti
◯ Dominica
◯ Dominican Republic
◯ DR Congo
◯ Ecuador
◯ Egypt
◯ El Salvador
◯ Equatorial Guinea
◯ Eritrea
◯ Estonia
◯ Eswatini
◯ Ethiopia
◯ Faeroe Islands
◯ Finland
◯ France
◯ French Guiana
◯ Gabon
◯ Gambia
◯ Georgia
◯ Germany
◯ Ghana
◯ Gibraltar
◯ Greece

◯ Grenada
◯ Guatemala
◯ Guinea
◯ Guinea-Bissau
◯ Guyana
◯ Haiti
◯ Holy See
◯ Honduras
◯ Hong Kong
◯ Hungary
◯ Iceland
◯ India
◯ Indonesia
◯ Iran
◯ Iraq
◯ Ireland
◯ Isle of Man
◯ Israel
◯ Italy
◯ Jamaica
◯ Japan
◯ Jordan
◯ Kazakhstan
◯ Kenya
◯ Kuwait
◯ Kyrgyzstan
◯ Laos
◯ Latvia
◯ Lebanon
◯ Lesotho
◯ Liberia
◯ Libya
◯ Liechtenstein
◯ Lithuania
◯ Luxembourg
◯ Macao
◯ Madagascar
◯ Malawi
◯ Malaysia
◯ Maldives
◯ Mali
◯ Malta
◯ Mauritania
◯ Mauritius
◯ Mayotte
◯ Mexico
◯ Moldova
◯ Monaco
◯ Mongolia
◯ Montenegro
◯ Morocco
◯ Mozambique
◯ Myanmar
◯ Namibia
◯ Nepal
◯ Netherlands
◯ Nicaragua
◯ Niger
◯ Nigeria
◯ North Korea
◯ North Macedonia
◯ Norway
◯ Oman
◯ Pakistan
◯ Panama
◯ Paraguay
◯ Peru
◯ Philippines
◯ Poland
◯ Portugal
◯ Qatar

◯ Réunion
◯ Romania
◯ Russia
◯ Rwanda
◯ Saint Helena
◯ Saint Kitts and Nevis
◯ Saint Lucia
◯ Saint Vincent and the Grenadines
◯ San Marino
◯ Sao Tome & Principe
◯ Saudi Arabia
◯ Senegal
◯ Serbia
◯ Seychelles
◯ Sierra Leone
◯ Singapore
◯ Slovakia
◯ Slovenia
◯ Somalia
◯ South Africa
◯ South Korea
◯ South Sudan
◯ Spain
◯ Sri Lanka
◯ State of Palestine
◯ Sudan
◯ Suriname
◯ Sweden
◯ Switzerland
◯ Syria
◯ Taiwan
◯ Tajikistan
◯ Tanzania
◯ Thailand
◯ The Bahamas
◯ Timor-Leste
◯ Togo
◯ Trinidad and Tobago
◯ Tunisia
◯ Turkey
◯ Turkmenistan
◯ Uganda
◯ Ukraine
◯ United Arab Emirates
◯ United Kingdom
◯ United States
◯ Uruguay
◯ Uzbekistan
◯ Venezuela
◯ Vietnam
◯ Western Sahara
◯ Yemen
◯ Zambia
◯ Zimbabwe

What is your profession? (select all that apply)

- ☐ Nurse
- ☐ Clinical officer
- ☐ Medical officer
- ☐ General practitioner
- ☐ Family medicine specialist
- ☐ Internal medicine specialist
- ☐ Pediatrics specialist
- ☐ Infectious diseases specialist
- ☐ Pulmonology specialist
- ☐ Public health specialist
- ☐ Policy maker
- ☐ Pharmacist
- ☐ Other

Please specify: ______________________

Characterize your professional activities related to TB care: (select all that apply)

- ☐ I am a general health care provider and occasionally provide care for patients with or at risk for TB
- ☐ I provide specialist care for TB
- ☐ I train other providers on TB care and treatment
- ☐ I develop and/or implement guidelines for TB prevention and treatment
- ☐ I conduct research on TB prevention and treatment
- ☐ Other

Please specify: ______________________

How many years of experience do you have with TB preventive treatment?

- ◯ < 5y
- ◯ 5-10y
- ◯ 10-20y
- ◯ >20y

How many patients do you estimate you have treated for TB prevention in the last 12 months? ______________________

Are there national TB preventive treatment guidelines in your country?

- ◯ Yes
- ◯ No
- ◯ Unsure

Which treatment is recommended? (select all that apply)

- ☐ 6 - 9 months of daily isoniazid
- ☐ 4 months of daily rifampin
- ☐ 3 months of daily isoniazid with rifampin
- ☐ 3 months of weekly rifapentine with isoniazid
- ☐ 1 month of daily rifapentine with isoniazid
- ☐ Other

Please specify: ______________________

Do you prescribe TB preventive treatment?

◯ Yes
◯ No
◯ Unsure

How would you best characterize the setting in which you most frequently prescribe TB preventive treatment?

◯ Urban
◯ Rural
◯ Other
◯ N/A

Please specify: ______________________________

How would you best characterize the location(s) in which you prescribe TB preventive treatment? (select all that apply)

☐ Outpatient Clinic
☐ Hospital-based clinic
☐ Specialized center
☐ Community-based center
☐ Mobile clinic
☐ Prison or other incarceration setting
☐ Other

Please specify: ______________________________

Does your treatment location administer intramuscular injections for any medications?

◯ Yes
◯ No
◯ Unsure

Does your treatment location administer any of these other listed long-acting treatment formulations? (select all that apply)

☐ Contraceptive implants
☐ Prescription of medication patches
☐ None
☐ Other

Please specify: ______________________________

Including visits for diagnosis, treatment, and follow-up testing, how many visits does a patient make for the prevention of TB?

◯ 1-2
◯ 3-5
◯ Variable depending on risk factors
◯ Other

Please specify: ______________________________

Please indicate how often each of the following items is a BARRIER for patients taking TB preventive treatment. Please rate each of the following 1-5 (1 = not a barrier, 3 = sometimes a barrier, 5 = always a barrier)

	1 = Not a barrier	2	3 = Sometimes a barrier	4	5 = Always a barrier
Remembering to take oral pills	◯	◯	◯	◯	◯

Please indicate how often each of the following items is a BARRIER for patients taking TB preventive treatment. Please rate each of the following 1-5 (1 = not a barrier, 3 = sometimes a barrier, 5 = always a barrier)

Medications become lost ○ ○ ○ ○ ○

Please indicate how often each of the following items is a BARRIER for patients taking TB preventive treatment. Please rate each of the following 1-5 (1 = not a barrier, 3 = sometimes a barrier, 5 = always a barrier)

Food and/or Housing instability ○ ○ ○ ○ ○

Please indicate how often each of the following items is a BARRIER for patients taking TB preventive treatment. Please rate each of the following 1-5 (1 = not a barrier, 3 = sometimes a barrier, 5 = always a barrier)

Employment/work constraints ○ ○ ○ ○ ○

Please indicate how often each of the following items is a BARRIER for patients taking TB preventive treatment. Please rate each of the following 1-5 (1 = not a barrier, 3 = sometimes a barrier, 5 = always a barrier)

Stigma ○ ○ ○ ○ ○

Please indicate how often each of the following items is a BARRIER for patients taking TB preventive treatment. Please rate each of the following 1-5 (1 = not a barrier, 3 = sometimes a barrier, 5 = always a barrier)

Drug costs ○ ○ ○ ○ ○

Please indicate how often each of the following items is a BARRIER for patients taking TB preventive treatment. Please rate each of the following 1-5 (1 = not a barrier, 3 = sometimes a barrier, 5 = always a barrier)

Other related medical costs ○ ○ ○ ○ ○

Please indicate how often each of the following items is a BARRIER for patients taking TB preventive treatment. Please rate each of the following 1-5 (1 = not a barrier, 3 = sometimes a barrier, 5 = always a barrier)

Transport limitations, distance, or frequency for picking up medications ○ ○ ○ ○ ○

Please indicate how often each of the following items is a BARRIER for patients taking TB preventive treatment. Please rate each of the following 1-5 (1 = not a barrier, 3 = sometimes a barrier, 5 = always a barrier)

Side effects ○ ○ ○ ○ ○

Please indicate how often each of the following items is a BARRIER for patients taking TB preventive treatment. Please rate each of the following 1-5 (1 = not a barrier, 3 = sometimes a barrier, 5 = always a barrier)

Other	○	○	○	○	○

Please specify: ______________________________

Please rate the importance of each of the following FACTORS THAT WOULD INFLUENCE your decision to prescribe a long-acting medication for TB prevention rather than oral medications? Please rate the importance of each of these aspects from 1-5 (1 = not important, 3 = somewhat important, 5 = very important)

	1 = Not important	2	3 = Somewhat important	4	5 = Very important
Better efficacy	○	○	○	○	○

Please rate the importance of each of the following FACTORS THAT WOULD INFLUENCE your decision to prescribe a long-acting medication for TB prevention rather than oral medications? Please rate the importance of each of these aspects from 1-5 (1 = not important, 3 = somewhat important, 5 = very important)

Less side effects	○	○	○	○	○

Please rate the importance of each of the following FACTORS THAT WOULD INFLUENCE your decision to prescribe a long-acting medication for TB prevention rather than oral medications? Please rate the importance of each of these aspects from 1-5 (1 = not important, 3 = somewhat important, 5 = very important)

Lower cost to patient	○	○	○	○	○

Please rate the importance of each of the following FACTORS THAT WOULD INFLUENCE your decision to prescribe a long-acting medication for TB prevention rather than oral medications? Please rate the importance of each of these aspects from 1-5 (1 = not important, 3 = somewhat important, 5 = very important)

Lower cost to health system	○	○	○	○	○

Please rate the importance of each of the following FACTORS THAT WOULD INFLUENCE your decision to prescribe a long-acting medication for TB prevention rather than oral medications? Please rate the importance of each of these aspects from 1-5 (1 = not important, 3 = somewhat important, 5 = very important)

Fewer clinical visits	○	○	○	○	○

Please rate the importance of each of the following FACTORS THAT WOULD INFLUENCE your decision to prescribe a long-acting medication for TB prevention rather than oral medications? Please rate the importance of each of these aspects from 1-5 (1 = not important, 3 = somewhat important, 5 = very important)

Less health system infrastructure or clinical personnel required ○ ○ ○ ○ ○

Please rate the importance of each of the following FACTORS THAT WOULD INFLUENCE your decision to prescribe a long-acting medication for TB prevention rather than oral medications? Please rate the importance of each of these aspects from 1-5 (1 = not important, 3 = somewhat important, 5 = very important)

Improved adherence ○ ○ ○ ○ ○

Please rate the importance of each of the following FACTORS THAT WOULD INFLUENCE your decision to prescribe a long-acting medication for TB prevention rather than oral medications? Please rate the importance of each of these aspects from 1-5 (1 = not important, 3 = somewhat important, 5 = very important)

Decreased TB spread in the community ○ ○ ○ ○ ○

Please rate the importance of each of the following FACTORS THAT WOULD INFLUENCE your decision to prescribe a long-acting medication for TB prevention rather than oral medications? Please rate the importance of each of these aspects from 1-5 (1 = not important, 3 = somewhat important, 5 = very important)

Improved patient satisfaction or quality of life ○ ○ ○ ○ ○

Please rate the importance of each of the following FACTORS THAT WOULD INFLUENCE your decision to prescribe a long-acting medication for TB prevention rather than oral medications? Please rate the importance of each of these aspects from 1-5 (1 = not important, 3 = somewhat important, 5 = very important)

Recommendation by local treatment guidelines ○ ○ ○ ○ ○

Please rate the importance of each of the following FACTORS THAT WOULD INFLUENCE your decision to prescribe a long-acting medication for TB prevention rather than oral medications? Please rate the importance of each of these aspects from 1-5 (1 = not important, 3 = somewhat important, 5 = very important)

Recommendation by global treatment guidelines ○ ○ ○ ○ ○

Please rate the importance of each of the following FACTORS THAT WOULD INFLUENCE your decision to prescribe a long-acting medication for TB prevention rather than oral medications? Please rate the importance of each of these aspects from 1-5 (1 = not important, 3 = somewhat important, 5 = very important)

Recommended by my peers and/or professional society	◯	◯	◯	◯	◯

Please rate the importance of each of the following FACTORS THAT WOULD INFLUENCE your decision to prescribe a long-acting medication for TB prevention rather than oral medications? Please rate the importance of each of these aspects from 1-5 (1 = not important, 3 = somewhat important, 5 = very important)

More revenue or reimbursement to my clinic	◯	◯	◯	◯	◯

Please rate the importance of each of the following FACTORS THAT WOULD INFLUENCE your decision to prescribe a long-acting medication for TB prevention rather than oral medications? Please rate the importance of each of these aspects from 1-5 (1 = not important, 3 = somewhat important, 5 = very important)

Patient preference	◯	◯	◯	◯	◯

Please indicate for each of the following PATIENT CHARACTERISTICS if you would be less or more likely to prescribe a long-acting medication rather than oral medication(1 = less likely to prescribe long-acting medication, 3 = neither less or more likely, 5 = more likely to prescribe long-acting medication)

	1 = Less likely to prescribe long-acting medication	2	3 = Neither less or more likely to prescribe long-acting medication	4	5 = More likely to prescribe long-acting medication
Patient has HIV co-infection	◯	◯	◯	◯	◯

Please indicate for each of the following PATIENT CHARACTERISTICS if you would be less or more likely to prescribe a long-acting medication rather than oral medication(1 = less likely to prescribe long-acting medication, 3 = neither less or more likely, 5 = more likely to prescribe long-acting medication)

Patient has other chronic co-morbidities	◯	◯	◯	◯	◯

Please indicate for each of the following PATIENT CHARACTERISTICS if you would be less or more likely to prescribe a long-acting medication rather than oral medication(1 = less likely to prescribe long-acting medication, 3 = neither less or more likely, 5 = more likely to prescribe long-acting medication)

Patient has severe liver disease or cirrhosis ○ ○ ○ ○ ○

Please indicate for each of the following PATIENT CHARACTERISTICS if you would be less or more likely to prescribe a long-acting medication rather than oral medication(1 = less likely to prescribe long-acting medication, 3 = neither less or more likely, 5 = more likely to prescribe long-acting medication)

Patient has unstable housing ○ ○ ○ ○ ○

Please indicate for each of the following PATIENT CHARACTERISTICS if you would be less or more likely to prescribe a long-acting medication rather than oral medication(1 = less likely to prescribe long-acting medication, 3 = neither less or more likely, 5 = more likely to prescribe long-acting medication)

Patient abuses alcohol ○ ○ ○ ○ ○

Please indicate for each of the following PATIENT CHARACTERISTICS if you would be less or more likely to prescribe a long-acting medication rather than oral medication(1 = less likely to prescribe long-acting medication, 3 = neither less or more likely, 5 = more likely to prescribe long-acting medication)

Patient does not routinely engage in medical care ○ ○ ○ ○ ○

Please indicate for each of the following PATIENT CHARACTERISTICS if you would be less or more likely to prescribe a long-acting medication rather than oral medication(1 = less likely to prescribe long-acting medication, 3 = neither less or more likely, 5 = more likely to prescribe long-acting medication)

Patient comes from a poor or marginalized socioeconomic or ethnic group ○ ○ ○ ○ ○

Please indicate for each of the following PATIENT CHARACTERISTICS if you would be less or more likely to prescribe a long-acting medication rather than oral medication(1 = less likely to prescribe long-acting medication, 3 = neither less or more likely, 5 = more likely to prescribe long-acting medication)

Patient is currently incarcerated ○ ○ ○ ○ ○

Please indicate for each of the following PATIENT CHARACTERISTICS if you would be less or more likely to prescribe a long-acting medication rather than oral medication(1 = less likely to prescribe long-acting medication, 3 = neither less or more likely, 5 = more likely to prescribe long-acting medication)

Patient is elderly ◯ ◯ ◯ ◯ ◯

Please indicate for each of the following PATIENT CHARACTERISTICS if you would be less or more likely to prescribe a long-acting medication rather than oral medication(1 = less likely to prescribe long-acting medication, 3 = neither less or more likely, 5 = more likely to prescribe long-acting medication)

Patient is a child or adolescent ◯ ◯ ◯ ◯ ◯

If approved, and efficacy, safety, and cost were the same, would you prescribe a long-acting medication for TB prevention rather than oral medication?

◯ Yes, for all patients
◯ Yes, only for patients with certain characteristics or at high risk for poor adherence or who may prefer long-acting medications
◯ No
◯ Maybe/unsure
◯ Other

Please specify: __________________________________

If efficacy, safety, and cost were the same as an oral regimen, how likely would you be to prescribe each of the following modalities? (1-5: 1 = unlikely, 3 = somewhat likely, 5 = very likely)

	1 = Unlikely	2	3 = Somewhat likely	4	5 = Very likely
Injection (assume a one-time injection in the arm)	◯	◯	◯	◯	◯

If efficacy, safety, and cost were the same as an oral regimen, how likely would you be to prescribe each of the following modalities? (1-5: 1 = unlikely, 3 = somewhat likely, 5 = very likely)

Implant ◯ ◯ ◯ ◯ ◯

If efficacy, safety, and cost were the same as an oral regimen, how likely would you be to prescribe each of the following modalities? (1-5: 1 = unlikely, 3 = somewhat likely, 5 = very likely)

Patch ◯ ◯ ◯ ◯ ◯

If efficacy, safety, and cost were the same, which modality would you most prefer to prescribe?

◯ Oral pills
◯ Injection
◯ Implant
◯ Patch

Please select the response that best characterizes your view on the cost to the patient of a long-acting medication (Yes, No, Unsure).

I would prescribe a long-acting medication if compared to oral medications it costs to the patient...

	Yes	No	Unsure
...less or same	○	○	○

Please select the response that best characterizes your view on the cost to the patient of a long-acting medication (Yes, No, Unsure).

I would prescribe a long-acting medication if compared to oral medications it costs to the patient...

...twice as much	○	○	○

Please select the response that best characterizes your view on the cost to the patient of a long-acting medication (Yes, No, Unsure).

I would prescribe a long-acting medication if compared to oral medications it costs to the patient...

...three times as much	○	○	○

Please select the response that best characterizes your view on the cost to the patient of a long-acting medication (Yes, No, Unsure).

I would prescribe a long-acting medication if compared to oral medications it costs to the patient...

...regardless of cost	○	○	○

Please rate each of the following possible CONCERNS in prescribing a long-acting INJECTION in your context? Please rate each of these potential concerns 1-5 (1 = not a concern, 3 = might be a concern, 5 = definitely a concern)

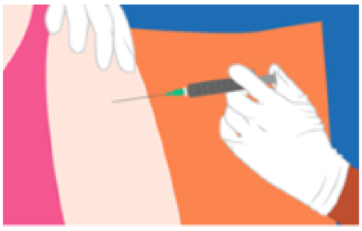

	1 = Not a concern	2	3 = Might be a concern	4	5 = Definitely a concern
Injection pain to the patient	○	○	○	○	○
Need for multiple injections	○	○	○	○	○
Unexpected side effects	○	○	○	○	○

Side effects lasting longer	◯	◯	◯	◯	◯
Difficult to administer	◯	◯	◯	◯	◯
Lower efficacy	◯	◯	◯	◯	◯
Interactions with other medications	◯	◯	◯	◯	◯

Regarding a long-acting INJECTION for TB prevention, HOW LIKELY would you be to prescribe the medication compared to current oral treatment in the following scenarios: (1 = unlikely, 3 = somewhat likely, 5 = very likely)

	1 = Unlikely	2	3 = Somewhat likely	4	5 =Very likely
Frequency of medication dosing: One-time medication dosing	◯	◯	◯	◯	◯

Regarding a long-acting INJECTION for TB prevention, HOW LIKELY would you be to prescribe the medication compared to current oral treatment in the following scenarios: (1 = unlikely, 3 = somewhat likely, 5 = very likely)

Frequency of medication dosing:<br><br>Monthly medication dosing for two months (2 doses total)	◯	◯	◯	◯	◯

Regarding a long-acting INJECTION for TB prevention, HOW LIKELY would you be to prescribe the medication compared to current oral treatment in the following scenarios: (1 = unlikely, 3 = somewhat likely, 5 = very likely)

Frequency of medication dosing:<br><br>Monthly medication dosing for three months (3 doses total)	◯	◯	◯	◯	◯

Overall, how feasible would it be to administer a long-acting INJECTION for TB prevention?

◯ 1 = Not feasible
◯ 2
◯ 3 = Somewhat feasible
◯ 4
◯ 5 =Very feasible

Please rate each of the following possible CONCERNS in prescribing a long-acting TB implant in your context? Please rate each of these potential concerns 1-5 (1 = not a concern, 3 = might be a concern, 5 = definitely a concern)

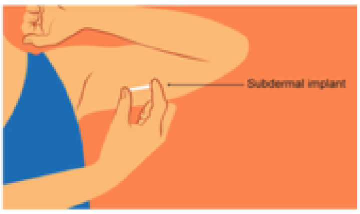

	1 = Not a concern	2	3 = Might be a concern	4	5 = Definitely a concern
Insertion site pain	◯	◯	◯	◯	◯
Scarring	◯	◯	◯	◯	◯
Other side effects	◯	◯	◯	◯	◯
Side effects lasting longer	◯	◯	◯	◯	◯
The implant may be visible to others (stigma)	◯	◯	◯	◯	◯
Difficult to administer	◯	◯	◯	◯	◯
Lower efficacy	◯	◯	◯	◯	◯
Interactions with other medications	◯	◯	◯	◯	◯
Unfamiliarity with the implant method of administration	◯	◯	◯	◯	◯

Overall, how feasible would it be to administer a long-acting IMPLANT for TB prevention?

◯ 1 = Not feasible
◯ 2
◯ 3 = Somewhat feasible
◯ 4
◯ 5 =Very feasible

Please rate each of the following possible CONCERNS in prescribing a long-acting TB PATCH in your context? Please rate each of these potential concerns 1-5 (1 = not a concern, 3 = might be a concern, 5 = definitely a concern)

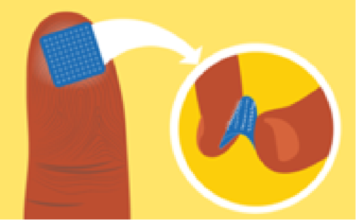

	1 = Not a concern	2	3 = Might be a concern	4	5 = Definitely a concern
Pain at the patch site	◯	◯	◯	◯	◯
Rash at the patch site	◯	◯	◯	◯	◯
Other potential side effects	◯	◯	◯	◯	◯
Side effects lasting longer than for oral drugs	◯	◯	◯	◯	◯
The patch may be visible to others (stigma)	◯	◯	◯	◯	◯
Difficult to administer	◯	◯	◯	◯	◯
Lower efficacy	◯	◯	◯	◯	◯
Interactions with other medications	◯	◯	◯	◯	◯

Unfamiliarity with the patch method of administration	◯	◯	◯	◯	◯

Overall, how feasible would it be to administer a long-acting PATCH for TB prevention?

◯ 1 = Not feasible
◯ 2
◯ 3 = Somewhat feasible
◯ 4
◯ 5 =Very feasible

How likely do you think each of the following factors are to influence your national government or health system to introduce long-acting medications for TB prevention in national guidelines and/or formularies? Please rate each of the following 1-5 (1 = not likely to influence, 3 = somewhat likely to influence, 5 = very likely to influence)

	1 = Not likely to influence	2	3 = Somewhat likely to influence	4	5 = Very likely to influence
Better efficacy	◯	◯	◯	◯	◯

How likely do you think each of the following factors are to influence your national government or health system to introduce long-acting medications for TB prevention in national guidelines and/or formularies? Please rate each of the following 1-5 (1 = not likely to influence, 3 = somewhat likely to influence, 5 = very likely to influence)

Less side effects	◯	◯	◯	◯	◯

How likely do you think each of the following factors are to influence your national government or health system to introduce long-acting medications for TB prevention in national guidelines and/or formularies? Please rate each of the following 1-5 (1 = not likely to influence, 3 = somewhat likely to influence, 5 = very likely to influence)

Lower cost to patient	◯	◯	◯	◯	◯

How likely do you think each of the following factors are to influence your national government or health system to introduce long-acting medications for TB prevention in national guidelines and/or formularies? Please rate each of the following 1-5 (1 = not likely to influence, 3 = somewhat likely to influence, 5 = very likely to influence)

Lower cost to health system	◯	◯	◯	◯	◯

How likely do you think each of the following factors are to influence your national government or health system to introduce long-acting medications for TB prevention in national guidelines and/or formularies? Please rate each of the following 1-5 (1 = not likely to influence, 3 = somewhat likely to influence, 5 = very likely to influence)

Fewer clinical visits	◯	◯	◯	◯	◯

How likely do you think each of the following factors are to influence your national government or health system to introduce long-acting medications for TB prevention in national guidelines and/or formularies? Please rate each of the following 1-5 (1 = not likely to influence, 3 = somewhat likely to influence, 5 = very likely to influence)

Less health system infrastructure or clinical personnel required ○ ○ ○ ○ ○

How likely do you think each of the following factors are to influence your national government or health system to introduce long-acting medications for TB prevention in national guidelines and/or formularies? Please rate each of the following 1-5 (1 = not likely to influence, 3 = somewhat likely to influence, 5 = very likely to influence)

Simplification of guidelines and/or formularies ○ ○ ○ ○ ○

How likely do you think each of the following factors are to influence your national government or health system to introduce long-acting medications for TB prevention in national guidelines and/or formularies? Please rate each of the following 1-5 (1 = not likely to influence, 3 = somewhat likely to influence, 5 = very likely to influence)

Improved adherence ○ ○ ○ ○ ○

How likely do you think each of the following factors are to influence your national government or health system to introduce long-acting medications for TB prevention in national guidelines and/or formularies? Please rate each of the following 1-5 (1 = not likely to influence, 3 = somewhat likely to influence, 5 = very likely to influence)

Decreased TB spread in the community ○ ○ ○ ○ ○

How likely do you think each of the following factors are to influence your national government or health system to introduce long-acting medications for TB prevention in national guidelines and/or formularies? Please rate each of the following 1-5 (1 = not likely to influence, 3 = somewhat likely to influence, 5 = very likely to influence)

Improved patient satisfaction or quality of life ○ ○ ○ ○ ○

How likely do you think each of the following factors are to influence your national government or health system to introduce long-acting medications for TB prevention in national guidelines and/or formularies? Please rate each of the following 1-5 (1 = not likely to influence, 3 = somewhat likely to influence, 5 = very likely to influence)

Recommendation by global treatment guidelines ○ ○ ○ ○ ○

How likely do you think each of the following factors are to influence your national government or health system to introduce long-acting medications for TB prevention in national guidelines and/or formularies? Please rate each of the following 1-5 (1 = not likely to influence, 3 = somewhat likely to influence, 5 = very likely to influence)

More revenue or reimbursement	○	○	○	○	○

How likely do you think each of the following factors are to influence your national government or health system to introduce long-acting medications for TB prevention in national guidelines and/or formularies? Please rate each of the following 1-5 (1 = not likely to influence, 3 = somewhat likely to influence, 5 = very likely to influence)

Patient preference	○	○	○	○	○

If the efficacy, safety, and cost of a long-acting medication were the same as the current oral regimen, HOW LIKELY do you believe your national authority would be to include long-acting medications into each of the following policies or processes? Please rate each 1-5 (1 = not likely, 3 = somewhat likely, 5 = very likely)

	1 = Not likely	2	3 = Somewhat likely	4	5 = Very likely
National drug approval and regulatory processes	○	○	○	○	○

If the efficacy, safety, and cost of a long-acting medication were the same as the current oral regimen, HOW LIKELY do you believe your national authority would be to include long-acting medications into each of the following policies or processes? Please rate each 1-5 (1 = not likely, 3 = somewhat likely, 5 = very likely)

National drug formulary	○	○	○	○	○

If the efficacy, safety, and cost of a long-acting medication were the same as the current oral regimen, HOW LIKELY do you believe your national authority would be to include long-acting medications into each of the following policies or processes? Please rate each 1-5 (1 = not likely, 3 = somewhat likely, 5 = very likely)

National TB prevention guidelines	○	○	○	○	○

Please rate each of the following potential OBSTACLES TO ADDRESS regarding introduction of long-acting medications for TB prevention in your country? Please rate each of the following 1-5 (1 = not an obstacle 3 = somewhat an obstacle 5 = likely an obstacle)

	1 = Not an obstacle	2	3 = Somewhat an obstacle	4	5 = Likely an obstacle
Drug approval regulatory processes	○	○	○	○	○

Please rate each of the following potential OBSTACLES TO ADDRESS regarding introduction of long-acting medications for TB prevention in your country? Please rate each of the following 1-5 (1 = not an obstacle 3 = somewhat an obstacle 5 = likely an obstacle)

Storage and distribution requirements ○ ○ ○ ○ ○

Please rate each of the following potential OBSTACLES TO ADDRESS regarding introduction of long-acting medications for TB prevention in your country? Please rate each of the following 1-5 (1 = not an obstacle 3 = somewhat an obstacle 5 = likely an obstacle)

Staffing for administration of injections ○ ○ ○ ○ ○

Please rate each of the following potential OBSTACLES TO ADDRESS regarding introduction of long-acting medications for TB prevention in your country? Please rate each of the following 1-5 (1 = not an obstacle 3 = somewhat an obstacle 5 = likely an obstacle)

Needle or syringe availability ○ ○ ○ ○ ○

Please rate each of the following potential OBSTACLES TO ADDRESS regarding introduction of long-acting medications for TB prevention in your country? Please rate each of the following 1-5 (1 = not an obstacle 3 = somewhat an obstacle 5 = likely an obstacle)

Cost of drugs ○ ○ ○ ○ ○

Please rate each of the following potential OBSTACLES TO ADDRESS regarding introduction of long-acting medications for TB prevention in your country? Please rate each of the following 1-5 (1 = not an obstacle 3 = somewhat an obstacle 5 = likely an obstacle)

Provider training and comfort ○ ○ ○ ○ ○

Please rate each of the following potential OBSTACLES TO ADDRESS regarding introduction of long-acting medications for TB prevention in your country? Please rate each of the following 1-5 (1 = not an obstacle 3 = somewhat an obstacle 5 = likely an obstacle)

Patient preferences or perceptions ○ ○ ○ ○ ○

Please rate each of the following potential OBSTACLES TO ADDRESS regarding introduction of long-acting medications for TB prevention in your country? Please rate each of the following 1-5 (1 = not an obstacle 3 = somewhat an obstacle 5 = likely an obstacle)

Concerns for side effects or drug interactions ○ ○ ○ ○ ○

Please specify side effect or drug interaction concern(s): ______________________________

Any other comments (or reasoning if survey is incomplete)?
